# Impact of mapped EQ-5D utilities on cost-effectiveness analysis: in the case of dialysis treatments

**DOI:** 10.1007/s10198-018-0987-x

**Published:** 2018-06-14

**Authors:** Fan Yang, Nancy Devlin, Nan Luo

**Affiliations:** 1 Centre for Health Economics, University of York, Heslington, York, YO10 5DD UK; 2 Office of Health Economics, London, UK; 3 Saw Swee Hock School of Public Health, National University of Singapore, Singapore, Singapore

**Keywords:** Cost-effectiveness, Dialysis, EQ-5D, Mapping, SF-6D, I10

## Abstract

**Objectives:**

This study aimed to evaluate the performance of EQ-5D data mapped from SF-12 in terms of estimating cost effectiveness in cost-utility analysis (CUA). The comparability of SF-6D (derived from SF-12) was also assessed.

**Methods:**

Incremental quality-adjusted life years (QALYs) and incremental cost-effectiveness ratios (ICERs) were calculated based on two Markov models assessing the cost effectiveness of haemodialysis (HD) and peritoneal dialysis (PD) using utility values based on EQ-5D-5L, EQ-5D using three direct-mapping algorithms and two response-mapping algorithms (mEQ-5D), and SF-6D. Bootstrap method was used to estimate the 95% confidence interval (percentile method) of incremental QALYs and ICERs with 1000 replications for the utilities.

**Results:**

In both models, compared to the observed EQ-5D values, mEQ-5D values expressed much lower incremental QALYs (range − 14.9 to − 33.2%) and much higher ICERs (range 17.5 to 49.7%). SF-6D also estimated lower incremental QALYs (− 29.0 and − 14.9%) and higher ICERs (40.9 and 17.5%) than did the observed EQ-5D. The 95% confidence interval of incremental QALYs and ICERs confirmed the lower incremental QALYs and higher ICERs estimated using mEQ-5D and SF-6D.

**Conclusion:**

Compared to observed EQ-5D, EQ-5D mapped from SF-12 and SF-6D would under-estimate the QALYs gained in cost-utility analysis and thus lead to higher ICERs. It would be more sensible to conduct CUA studies using directly collected EQ-5D data and to designate one single preference-based measure as reference case in a jurisdiction to achieve consistency in healthcare decision-making.

**Electronic supplementary material:**

The online version of this article (10.1007/s10198-018-0987-x) contains supplementary material, which is available to authorized users.

## Introduction

Estimation of health utility and quality-adjusted life years (QALYs) is an important part of cost-utility analysis (CUA) in economic evaluation [1]. Health utility can be generated from several preference-based utility measures, of which the EuroQol 5-dimension (EQ-5D) is the most commonly used. It is the preferred instrument of National Institute for Health and Care Excellence (NICE) in England for QALY estimation [2] and also being recommended or accepted by health technology assessment (HTA) agencies of many other countries [3–5]. Where desirable utility data were not collected, there is a need to map EQ-5D data from other patient-reported outcome instruments [6]. The mapping approach has been endorsed by NICE [7] for use in cases where EQ-5D data are not available and is increasingly popular for the purpose of estimating QALYs in CUAs [6].

There are various mapping algorithms available. The “source” predictive measures used to map to EQ-5D could be condition-specific quality of life measures (such as EORTC QLQ-C30 for cancer patients [8]), generic quality of life measures (such as Short Form 12-item (SF-12) [9]), clinical indicators of disease severity (such as Psoriasis Area and Severity Index [10]), or a combination of these. Also, data can be mapped to either the EQ-5D utility values or the EQ-5D item responses [11]. There are currently no clear guidelines on the best mapping method to EQ-5D for QALY estimation; so when deciding which mapping algorithm should be used in a particular study, whether it could generate comparable utility and cost-effectiveness estimates as the primarily collected EQ-5D would be the main consideration.

In addition, where a HTA agency has not stated its recommendation for one preference-based measure, other valid and robust preference-based instruments might be acceptable. Similar to EQ-5D, Short Form 6-dimension (SF-6D) is also widely used to estimate health utility for calculating QALYs [12]. Great differences in utility estimates derived from SF-6D and EQ-5D have been shown to exist [13–15], but only few studies have examined whether SF-6D could lead to comparable cost-effectiveness estimates as the directly derived EQ-5D [16, 17].

Therefore, this study aimed to evaluate the performance of EQ-5D data derived from multiple mapping algorithms in terms of estimating QALY gains in CUAs. The evaluation was based on one cost-utility analysis study of haemodialysis (HD) and peritoneal dialysis (PD) for patients with end-stage renal disease (ESRD) [18]. The comparability of SF-6D was also assessed.

## Methods

### Decision analytic models

Two Markov models used in a previous CUA study [18] were re-run in the present study (see Supplementary Fig. 1 for model structure). Model 1 and model 2 were constructed for non-diabetic and diabetic patients separately using different parameter values based on Singaporean local data and a 10-year time horizon was used (see Supplementary Table 1 for model transition probabilities). The analysis took the societal perspective and costs were reported in 2015 Singapore dollars ($). Details were reported elsewhere [18].

### Quality of life data

A consecutive sample of 75 patients undergoing HD and 75 patients undergoing PD for at least 3 months were interviewed in a cross-sectional survey using a battery of questionnaires including 5-level EQ-5D (EQ-5D-5L), SF-12, disease-specific scales of the 36-item Kidney Disease Quality of Life questionnaire (KDQOL-36), and questions assessing socio-demographic characteristics [19].

The EQ-5D-5L self-report questionnaire has five items (mobility, self-care, usual activities, pain/discomfort, and anxiety/depression) [20], with five descriptive levels for each item. The five levels include “no problems”, “slight problems”, “moderate problems”, and “severe problems” for all five items, and “unable to do” for mobility, self-care and usual activities and “extreme problems” for pain/discomfort and anxiety/depression. EQ-5D-5L items assess respondents’ health status on the day of survey. The SF-12 is a commonly used generic health instrument including 12 items, with a 4-week recall period, producing two summary scores, physical component summary (PCS) and mental component summary (MCS) [21].

### Estimation of utilities

Individual-level utilities were generated through the following approaches. First, utilities were calculated from EQ-5D-5L data using the recently developed EQ-5D-5L value set in England [22]. Second, five mapping functions were used to generate EQ-5D values from SF-12, including three functions mapping directly to utility values [9, 23, 24] and two functions mapping to EQ-5D responses [11]. Mapping function a. was developed using data from a low-income and predominantly minority patient sample attending a community health centre in US while other four functions were developed using the EQ-5D and SF-12 data collected from a representative general population sample in US. The UK EQ-5D-3L value set was used in all functions. Ordinary least squares (OLS) regression equations were used to directly map SF-12 to EQ-5D values including adjusted PCS and MCS (centered on the sample mean) and their interaction terms in function a. [23], PCS, MCS, and their interaction terms in function b [9]. and PCS and MCS only in function c. [24]. Multinomial logit regressions were used to map SF-12 summary scores (function d.) and individuals SF-12 questions (function e.) onto EQ-5D responses, respectively [11]. The mapping-derived utilities are hereafter referred to as “mEQ-5D” values. Third, SF-6D values were generated using responses to seven of the SF-12 items and a recommended algorithm [25], which is based on a set of preference weights obtained from a sample of the general population in the UK. The main characteristics of these methods are summarised in Table 1.

**Table 1:** Methods to generate health utility values from the EQ-5D-5L and SF-12 surveys

Calculation methods	Author	Value set country	Sample size	Valuation method	Value range
EQ-5D-5L value set	Devlin et al.	England	912	Composite TTO & DCE	(− 0.285, 1)
SF-12 mapped EQ-5D-3L	Franks et al.	UK	240	Direct mapping^a^	(− 0.140, 0.930)
	Franks et al.	UK	12,988	Direct mapping^b^	(− 0.118, 0.980)
	Lawrence and Fleishman	UK	14,580	Direct mapping^c^	(− 0.131, 1)
	Gray et al.	UK	12,967	Response mapping^d^	(− 0.594, 1)
	Gray et al.	UK	12,967	Response mapping^e^	(− 0.594, 1)
SF-12 based SF-6D	Brazier and Roberts	UK	611	Standard gamble	(0.345, 1)

*EQ-5D-3L* 3-level EuroQol-5D, *SF-12* Short Form-12, *SF-6D* Short Form 6-dimension, *TTO* time trade-off, *DCE* discrete choice experiment

^a^PCS and MCS were centered on the sample mean and then included in ordinary least squares model with the interaction terms

^b^PCS, MCS, and the interaction terms were included in ordinary least squares model

^c^PCS and MCS were included in ordinary least squares model

^d^PCS, MCS, and the interaction terms were used in multinomial logit model

^e^Individual SF-12 questions were used in multinomial logit model

Using these individual-level utility values, multivariate linear regression models were run to predict the mean utility values for HD- and PD-treated non-diabetic and diabetic ESRD patients controlling for socio-demographic characteristics. The predicted mean utility values for HD and PD states were used in model 1 and model 2. Utility values for transplantation were obtained from a published meta-analysis [26]. Utilities values for all health states were assumed constant within the time horizon of both models.

### Analysis

In each model, a hypothetical cohort of 10,000 patients was modelled to estimate the incremental costs and QALYs gained from HD and PD for an average patient. Discounting at an annual rate of 3% was applied to both costs and QALYs. The incremental cost-effectiveness ratios (ICERs) of HD compared to PD were calculated for the two models separately. Difference in EQ-5D and mEQ-5D/SF-6D based incremental QALYs and ICERs was examined. To examine the variability in utility estimates for the two health states in both models, non-parametric bootstrap method was used to estimate the 95% confidence interval (percentile method) of incremental QALYs and ICERs with 1,000 replications [27, 28]. All analyses were performed using Microsoft Excel 2016.

## Results

**Fig. 1:**
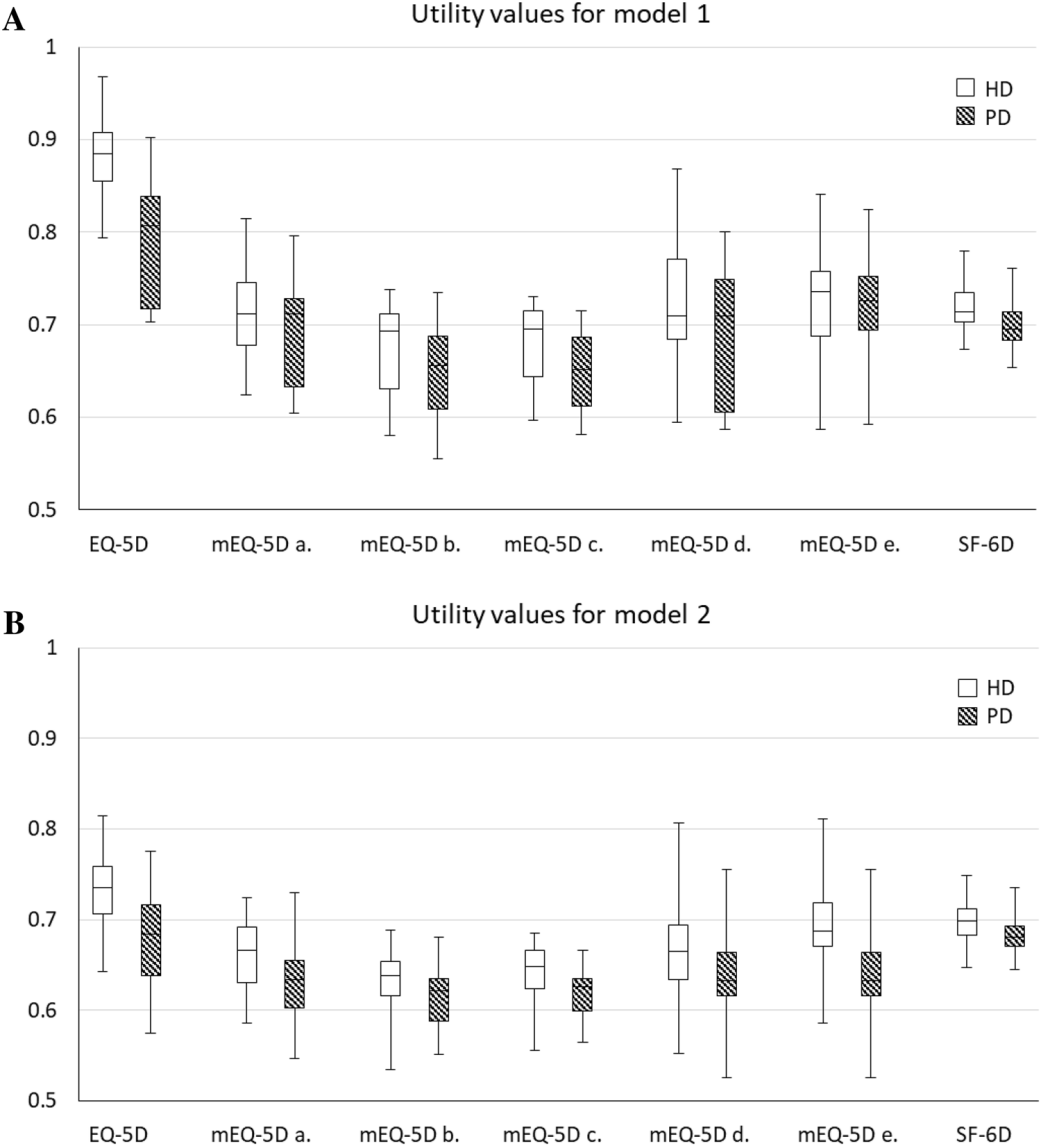
Box plots of utilities for HD and PD states used in model 1 (**a**) and model 2 (**b**)

Figure 1 presents the box plots of the utilities values for HD and PD states used in model 1 and model 2. In both models, patients on HD had higher utility values than those on PD. For both HD and PD, mEQ-5D generated lower values than EQ-5D and so did SF-6D. Among the mEQ-5D values, the error margins estimated by the response-mapping functions (i.e. function d. and e.) were wider than the direct-mapping functions. Table 2 summarises the mean utility values and utility differences between HD and PD states. The between-group utility differences varied with the approach used to generate utility values; the EQ-5D values exhibited much larger differences than the mEQ-5D and SF-6D values.

**Table 2:** Mean utility scores, between-group utility differences, incremental QALYs estimated using different methods

	Utilities			Incremental QALYs			ICER		
	HD	PD	Difference (HD-PD)	Mean	% Difference	Bootstrap 95% CI	Mean	% Difference	Bootstrap 95% CI
Model 1									
EQ-5D	0.882	0.803	0.079	2.011	–	1.891–2.138	59,073	–	55,564–62,822
mEQ-5D a.	0.714	0.701	0.013	1.384	− 31.2%	1.260–1.519	85,835	45.3%	78,207–94,283
mEQ-5D b.	0.676	0.658	0.018	1.343	− 33.2%	1.230–1.456	88,456	49.7%	81,591–96,582
mEQ-5D c.	0.683	0.655	0.027	1.399	− 30.4%	1.290–1.499	84,915	43.7%	79,250–92,090
mEQ-5D d.	0.721	0.709	0.012	1.392	− 30.8%	1.216–1.564	85,342	44.5%	75,957–97,694
mEQ-5D e.	0.725	0.723	0.002	1.348	− 33.0%	1.177–1.511	88,128	49.2%	78,621–100,931
SF-6D	0.718	0.698	0.020	1.427	− 29.0%	1.357–1.488	83,249	40.9%	79,836–87,543
Model 2									
EQ-5D	0.739	0.677	0.062	1.603	–	1.490–1.699	70,193	–	66,227–75,517
mEQ-5D a.	0.661	0.631	0.030	1.342	− 16.3%	1.255–1.425	83,845	19.4%	78,961–89,657
mEQ-5D b.	0.627	0.613	0.013	1.215	− 24.2%	1.123–1.289	92,609	31.9%	87,292–100,196
mEQ-5D c.	0.640	0.617	0.023	1.278	− 20.3%	1.198–1.343	88,044	25.4%	83,783–93,923
mEQ-5D d.	0.671	0.640	0.031	1.364	− 14.9%	1.248–1.501	82,493	17.5%	74,963–90,160
mEQ-5D e.	0.696	0.683	0.013	1.339	− 16.5%	1.241–1.451	84,033	19.7%	77,547–90,669
SF-6D	0.699	0.681	0.018	1.364	− 14.9%	1.315–1.423	82,493	17.5%	79,072–85,597

Bootstrap denotes the bootstrap percentile method with 1000 bootstrap replications

*CI* confidence interval, *HD* haemodialysis, *ICER* incremental cost-effectiveness ratio, *PD* peritoneal dialysis, *EQ-5D* EuroQol-5D, *SF-6D* Short Form 6-dimension

Table 2 also presents the estimated incremental QALYs. In model 1, incremental QALY was 2.011 using EQ-5D values, 1.343 to 1.399 using mEQ-5D values, and 1.427 using SF-6D values. The corresponding figures were 1.603, 1.215 to 1.364, and 1.364 in model 2. Compared to observed EQ-5D values, mapping algorithms generated much lower incremental QALYs (range − 14.9 to − 33.2%), with smallest differences using SF-12 summary scores to item responses mapping algorithm, i.e. function d (− 30.8% for model 1 and − 14.9% for model 2). The incremental QALYs estimated using SF-6D values were 29.0% lower for model 1 and 14.9% lower for model 2, compared to those estimated using observed EQ-5D values. The 95% confidence interval of incremental QALYs confirmed the lower incremental QALYs estimated using mapped EQ-5D and SF-6D than those estimated using observed EQ-5D (Table 2). The estimated ICERs are also summarised in Table 2. In both models, mEQ-5D generated much higher ICERs than observed EQ-5D (model 1, 43.7–49.7%; model 2, 17.5–25.4%) and so did SF-6D (model 1, 40.9%; model 2, 17.5%). The 95% confidence interval of ICERs also confirmed the higher estimated ICERs using mEQ-5D and SF-6D, compared to those using observed EQ-5D (Table 2).

## Discussion

This study observed substantially different results in incremental QALYs and ICERs estimated using mapped and directly captured EQ-5D data. Such results are not surprising as mapping technique has been found to introduce additional uncertainty into cost-effectiveness estimates and thus should be treated as a second best option [6]. First, the discrepancies could be explained by the considerable differences in the descriptive system of SF-12 and EQ-5D. Although both instruments are designed to measure some similar dimensions of health, their descriptive systems seem to capture different aspects of these dimensions [13, 29]. It is worth mentioning that the difference in recall period between the two instruments could also matter in this dialysis patient sample. The quality of life data for HD patients were collected while they were undergoing dialysis, and therefore, patients may take the effects of dialysis into consideration when assessing their own health on that day, as measured by EQ-5D, but consider the average health in the past 4 weeks when completing SF-12. As a result, the quality of life for HD patients may be over-estimated if measured using EQ-5D.

Second, the differences may also be due to a mismatch between the mapping functions and the study sample. The validity of mapping is based on the assumption that the statistical relationship is the same between the sample used to develop mapping functions and the target sample to which the mapping functions will be applied [6], so the mapping algorithm developed using data from patients whose characteristics were comparable to this dialysis sample would perform better in terms of validity. However, the currently available mapping functions (and used in this study) were estimated using either a low-income and minority patient sample or general population sample in US [30] which are much younger and healthier than the dialysis patient sample whose data are reported here [9, 11, 23]. The variations in results may imply that the mapping algorithms used in this study are not suitable for this dialysis patient sample. It is ideal to use the mapping functions based on data from dialysis patients, but such algorithm is not available yet.

Third, prediction bias is an inherent weakness of the mapping technique. OLS models used in direct mapping may not accurately predict the EQ-5D distribution for high values due to the ceiling effects of EQ-5D and over-predict utility values for patients in poor health [30]; the response-mapping approach could better reflect the distribution of EQ-5D, but no performance improvement was found [31, 32]. Interestingly, the SF-12 summary scores to EQ-5D response-mapping technique (mapping function d.) seems to perform best among the five algorithms, possibly because the SF-12 and EQ-5D could be better modelled using summary scores and item responses, respectively.

Last, the use of primary EQ-5D-5L data may also contribute to the discrepancies. The observed EQ-5D values were calculated using the EQ-5D-5L value set while all mapping functions were based on EQ-5D-3L values. Variations in estimating cost effectiveness using 3L and 5L value sets have been reported previously [33, 34]. However, due to the unavailability of primary EQ-5D-3L data, it is not possible to compare the results of mapped EQ-5D-3L values to those observed EQ-5D-3L values.

It should be noted that mapped EQ-5D values consistently generated much lower incremental QALYs and higher ICERs than directly captured EQ-5D values, which could be mainly driven by the much smaller between-group utility differences defined by mapped EQ-5D. As shown in a previous study [34] that incremental QALYs based on these two Markov models were a function of both utility of PD and difference in utilities of PD and HD; the performance of mapping algorithms in quantifying absolute utilities of the alternatives also contribute to the different results. The finding that mapped EQ-5D tends to generate fewer incremental QALYs and thus higher ICERs have important implications for decision-making in economic evaluation. It is possible that the technologies, which would be considered cost-effective using observed EQ-5D values may be rejected by the reimbursement agency if mapped EQ-5D data were used. Therefore, researchers and decision makers should be aware of the impact of using mapped utility estimates in economic evaluation. It is highly suggested that directly collected EQ-5D data should be used in CUAs to inform decision-making on new or existing health technologies.

When EQ-5D data are not available, but SF-12 data are, estimating health utility using SF-6D could be an alternative for countries without preference for a particular instrument. Our results show that SF-6D generated lower incremental QALY estimates and higher ICERs. A number of differences between SF-6D and EQ-5D could explain the differences, such as differences in the descriptive system [13, 29], valuation techniques (standard gamble used in SF-6D vs. time trade-off used in EQ-5D) [35, 36] and value ranges (0.345 to 1 in SF-6D vs. -0.594 to 1 in EQ-5D) [25]. This finding suggests that the cost-effectiveness results based on SF-6D are not identical to those based on EQ-5D and if the same willingness-to-pay threshold is applied, the reimbursement decisions based on SF-6D and EQ-5D values may be different. Therefore, for a HTA agency, it is better to designate one single preference-based measure as reference case to achieve consistency in decision-making.

This study is not without limitations. It is based on a single cost-effectiveness analysis of dialysis treatments in the context of Singapore, which undoubtedly limits the generalisability of its findings. Many previous studies including a variety of general population and patient samples also found that the smaller between-group utility differences estimated using mapped EQ-5D [37–39], same as this study. Another limitation is that the EQ-5D values were not calculated using the value set derived from the country where the original data were collected. Although the UK value sets were used consistently in both observed and mapped EQ-5D values to minimise the differences resulted from country-specific value sets, the applicability of UK value sets into Singaporean EQ-5D data may still be a concern.

## Conclusions

Compared to observed EQ-5D, mapped EQ-5D and SF-6D, would generate fewer QALY gains and higher ICERs in cost-utility analysis, which may lead to different conclusions about the cost effectiveness of health care. It would be more sensible to conduct CUA studies using directly collected EQ-5D data and to designate one single preference-based measure as reference case in a jurisdiction to achieve consistency in healthcare decision-making.

## Electronic supplementary material

Below is the link to the electronic supplementary material.

Supplementary material 1 (DOCX 90 KB)
