# Supplementary material for: Impact of mapped EQ-5D utilities on cost-effectiveness analysis: in the case of dialysis treatments
Source: Eur J Health Econ. 2018 Jun 14;20(1):99–105. doi: 10.1007/s10198-018-0987-x (PMC6394787; doi:10.1007/s10198-018-0987-x)
Supplement: Supplementary file 1 — Supplementary material 1 (DOCX 90 KB) [file 10198_2018_987_MOESM1_ESM.docx]

**
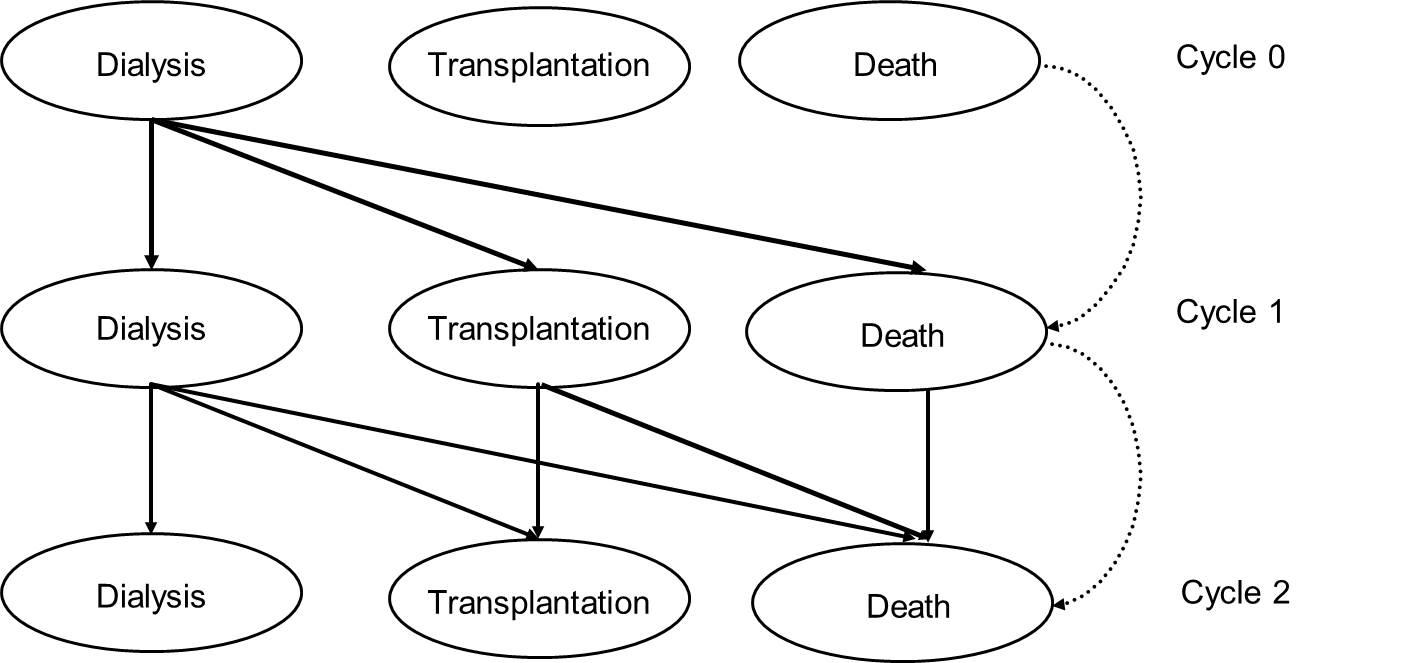
**

**Supplementary Figure 1.** Markov structure from cycle 0 to cycle 2. Each cycle represents a Markov state. Arrows indicate allowed transitions.

**Supplementary Table 1**. Summary of transition probabilities used in the two Markov models

|  | | **Definition** | | **Value** | **Source** | **Assumption** |
| --- | --- | --- | --- | --- | --- | --- |
| Model 1 (non-diabetic patients): | | HD-death | Year 1 | 0.031 | [1] | Transition probabilities were constant from the 5^th^ year onwards |
|  | |  | Year 2 | 0.033 |  |  |
|  | |  | Year 3 | 0.028 |  |  |
|  | |  | Year 4 | 0.020 |  |  |
|  | |  | Year 5 | 0.040 |  |  |
|  | | PD-death | Year 1 | 0.076 |  |  |
|  | |  | Year 2 | 0.093 |  |  |
|  | |  | Year 3 | 0.147 |  |  |
|  | |  | Year 4 | 0.082 |  |  |
|  | |  | Year 5 | 0.111 |  |  |
|  | | HD/PD-transplant |  | 0.018 | Singapore Renal Registry 2009 [2] |  |
|  | | Transplant-death |  | 0.021 |  |  |
|  | | HD-PD |  | 0 |  | No transition between HD and PD |
| Model 2 (diabetic patients): | | HD-death | Year 1 | 0.067 | [1] |  |
|  | |  | Year 2 | 0.083 |  |  |
|  | |  | Year 3 | 0.079 |  |  |
|  | |  | Year 4 | 0.040 |  |  |
|  | |  | Year 5 | 0.086 |  |  |
|  | | PD-death | Year 1 | 0.088 |  |  |
|  | |  | Year 2 | 0.136 |  |  |
|  | |  | Year 3 | 0.204 |  |  |
|  | |  | Year 4 | 0.187 |  |  |
|  | |  | Year 5 | 0.252 |  |  |
|  | | HD/PD-transplant |  | 0.018 | Singapore Renal Registry 2009 [2] |  |
|  | | Transplant-death |  | 0.021 |  |  |
|  | | HD-PD |  | 0 |  | No transition between HD and PD |
|  | [1] Yang F, Khin LW, Lau T, et al. Hemodialysis versus Peritoneal Dialysis: A Comparison of Survival Outcomes in South-East Asian Patients with End-Stage Renal Disease. *PLoS One.* 2015;10(10):e0140195.  [2] Choong HL. Eighth report of the Singapore renal registry 2009. 2012. | | | | | |
